# LOCC-GER - A georeferenced local climate policy database for Germany

**DOI:** 10.1038/s41597-026-08183-2

**Published:** 2026-10-07

**Authors:** Dennis Abel, Manuel Linsenmeier, Stefan Jünger, Amelie Veit

**Affiliations:** 1 GESIS - Leibniz Institute for the Social Sciences, Cologne, Germany; 2 Goethe University Frankfurt, Frankfurt am Main, Germany; 3 University of Bonn, Bonn, Germany

**Keywords:** Energy policy, Climate-change mitigation, Society

## Abstract

Local climate action has increased massively worldwide. Germany is a noteworthy case in this respect, as local measures have been strongly promoted by the federal system over the past two decades. However, studying policies, their determinants, and consequences at this scale has been difficult so far due to a lack of centralized data. This article introduces LOCC-GER, the largest existing dataset of georeferenced local climate policy-making in Germany. The dataset currently covers 26,142 measures from 5,033 municipalities, including detailed descriptions of employed measures, funding sum, and duration from 2008 to 2023. It is extended with important local context variables on public opinion and civil society activities, as well as detailed weather and climate indicators. We demonstrate how it can be used to map and analyze local governments’ actions on climate change. Finally, we discuss how LOCC-GER may be extended by academics and other users through our proposed workflow for user-specific linking with additional covariates, offering high spatial and temporal flexibility.

## Background & Summary

Local governments take on a central role in climate action in Germany’s multi-level system of government^1,2^. Germany’s decentralized, federalist system of government comprises 16 federal states (Bundesländer) and more than 10,752 municipalities (as of June 2024). Still, few sources of integrated data on policies and measures on these sub-levels exist. Regional data are often stored in separate accounts for each federal state, and local data are usually not systematically integrated and curated by public bodies. Furthermore, government releases on local-level data are primarily structural and administrative. They often lack in-depth indicators on policies and associated metrics, such as spending, instrument type, and domain. This lack of data has severely limited the opportunities for scholars to investigate this level of policy-making. While recent advances in data availability have primarily driven research at the national and state levels^3^, most studies of local climate policy rely on case study data (e.g.,^2^).

**Fig. 1:**
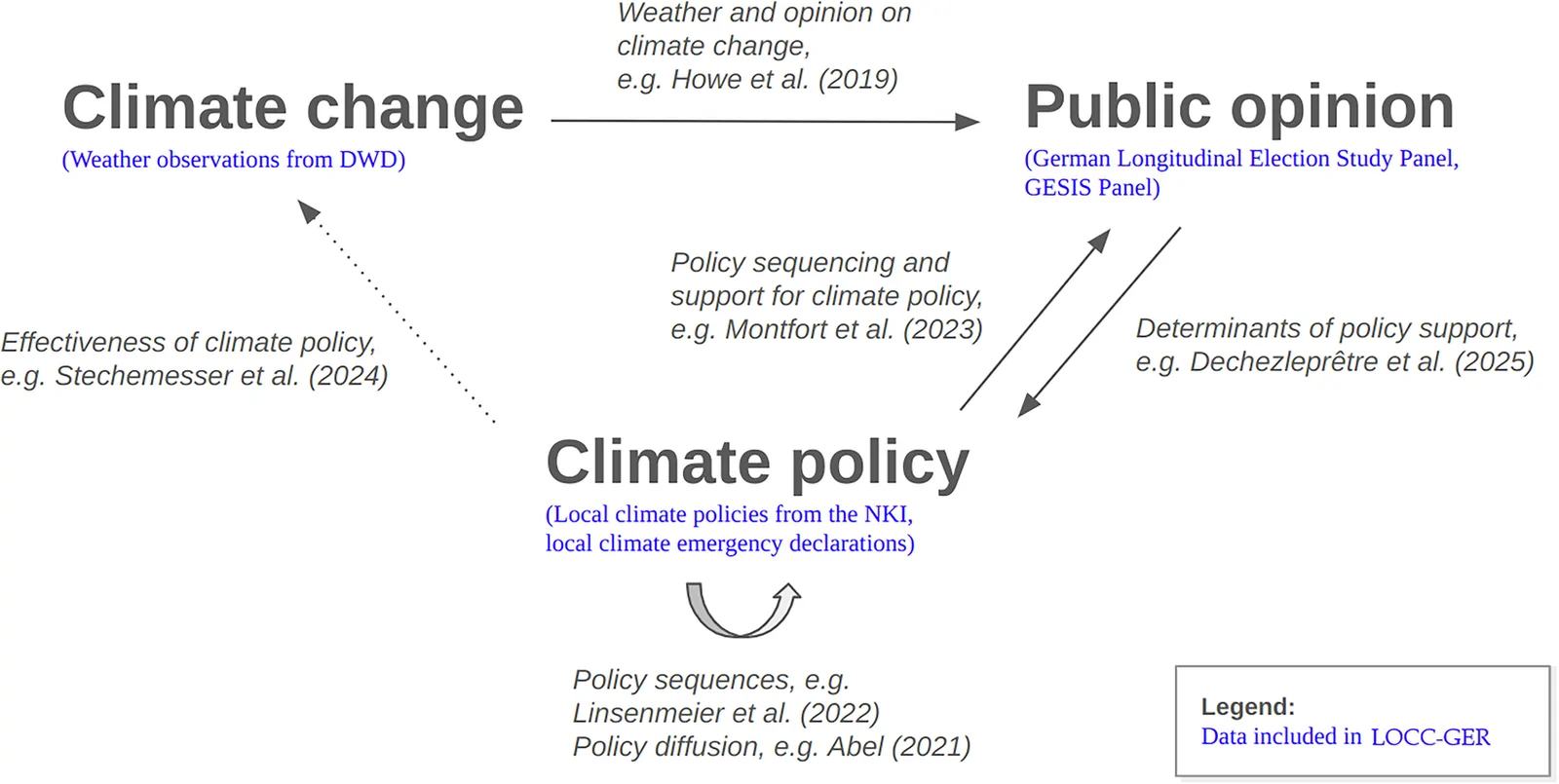
The climate policy loop.

It is at the local level, however, where much of the variation in climate action can be observed. This variation, in turn, offers rich opportunities for empirical research on the determinants of local climate policy and its consequences. This includes the often local effects of extreme weather events and climate change on public opinion^4^, the effect of public opinion on climate policy-making^5^ and vice versa^6^, as well as policy sequences^7,8^ and policy diffusion^9,10^ (Fig. 1). Indeed, recent research suggests that global support for climate policy varies as much within countries as between them^11^, motivating further work on the political economy of climate policy at the local level.

**Fig. 2:**
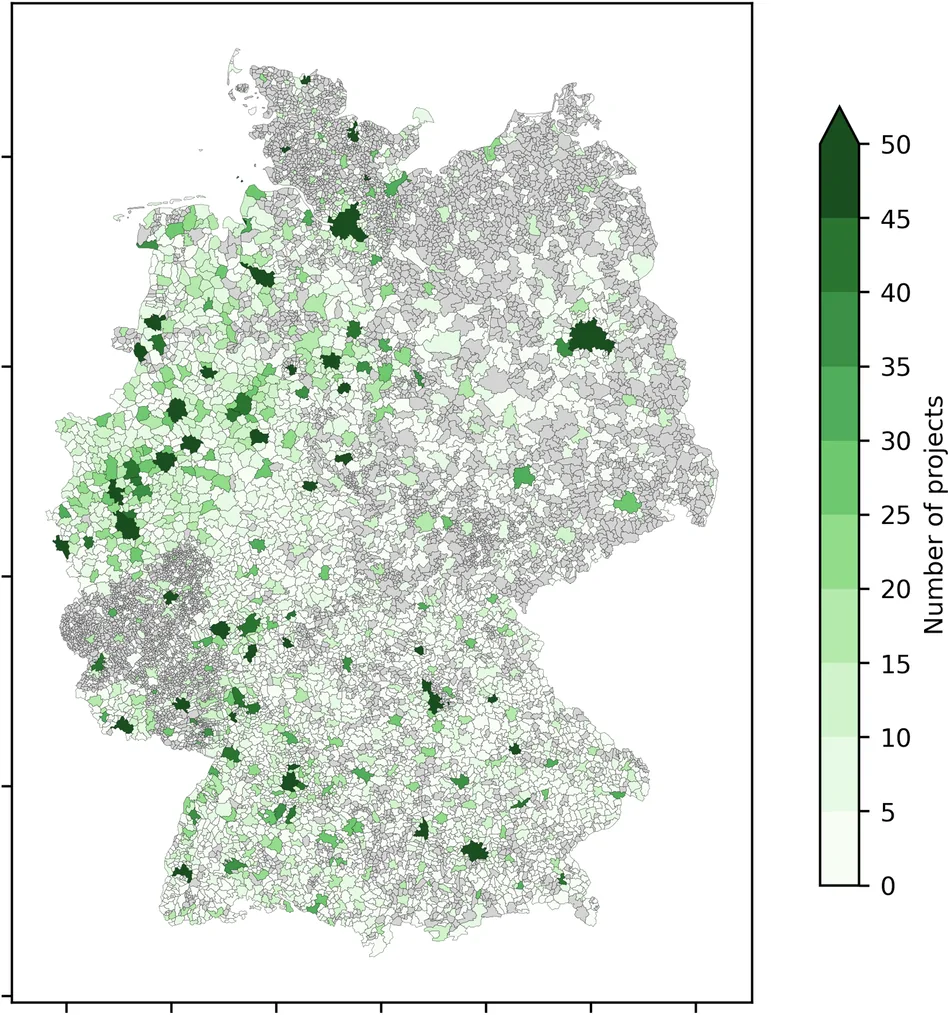
Number of climate policy projects by municipality.

This article introduces LOCC-GER to support the study of local climate action. LOCC-GER is one of the largest and most detailed datasets on municipal-level policy-making, making it of interest to public policy scholars beyond the scope of climate policy. Given the integration of four types of georeferenced variables: 1. Local climate policy, 2. civil society action, 3. climate indicators, and 4. public opinion data, it represents a valuable resource for topics such as local politics, climate change mitigation, and adaptation, as well as political movements. Concerning the policy content, the dataset offers unprecedented depth and detail. With over 25,000 policy records from more than 5,000 municipalities, users can explore site-specific climate action, including details on the exact measures and funding levels. The dataset is also unique in terms of temporal coverage. The records cover all years from 2008 to 2023, representing the most active period in German local climate policy-making. Fig. 2 maps the cumulative count of these projects in German municipalities. Finally, given our extensive data integration strategy described below, users are enabled to set up a linking workflow to georeferenced public opinion data and have access to crucial context variables, such as weather and climate patterns, for the entire study period.

Given researchers’ needs for up-to-date, customizable indicators tailored to the specific operationalization of their research questions, we offer extensive workflows for flexible data integration with crucial context factors, such as climate indicators, socio-economic factors, and public opinion, while adhering to Open Science and FAIR data principles. Furthermore, LOCC-GER is designed as a living dataset with annual updates to the policy database and an increasing geographical coverage. In the rest of the article, we provide a complete description of our data collection, processing, and validation, demonstrate example analyses, and close with a discussion of other use cases.

## Methods

**Fig. 3:**
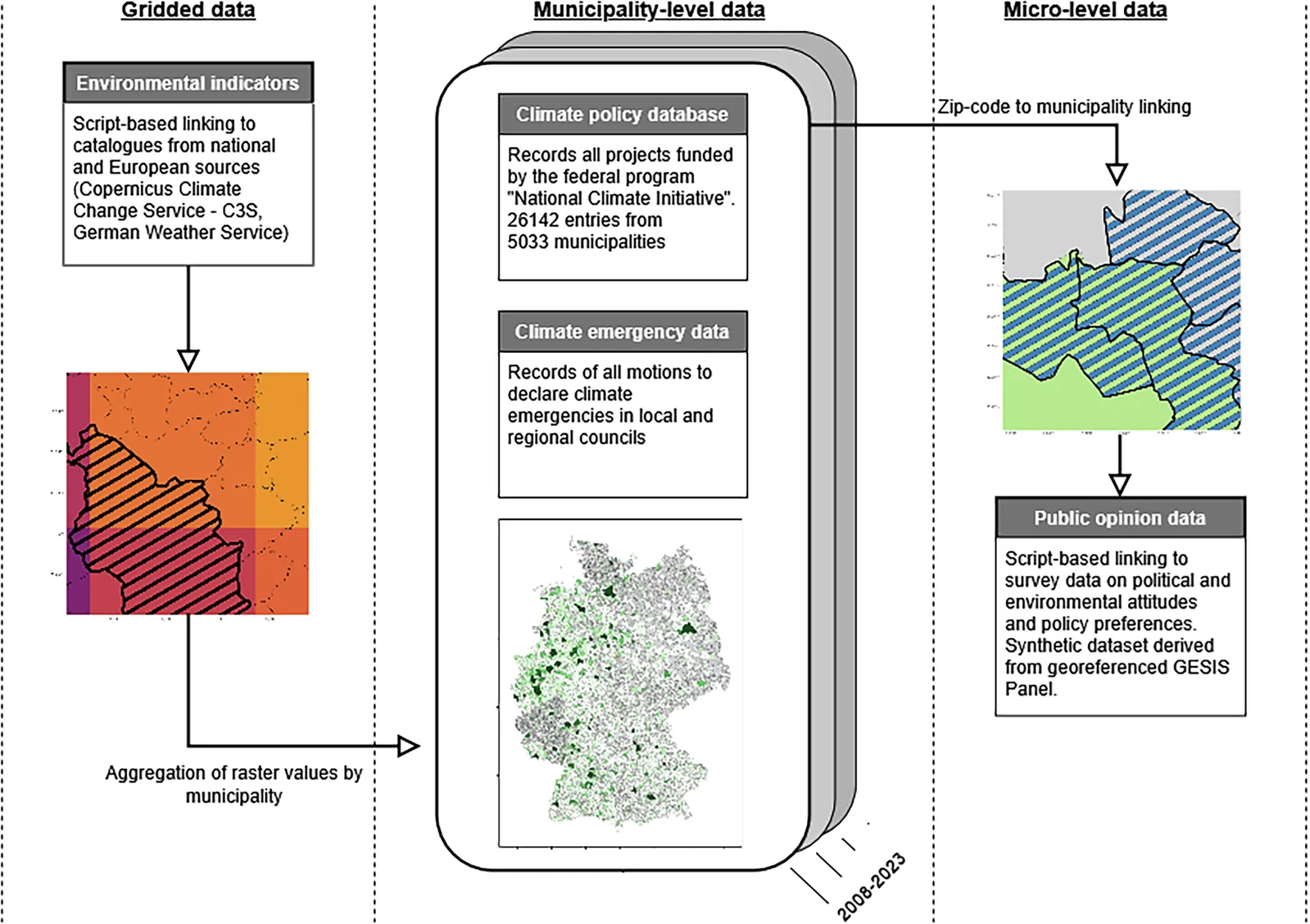
Data integration process.

Our dataset relies on a comprehensive and novel integration of several data sources (see Fig. 3). The core of the dataset comprises all projects funded by the federal scheme “National Climate Initiative” (NKI) between 2008 and 2023. This funding scheme represents the largest and most relevant federal approach to supporting and steering local climate action (see https://www.klimaschutz.de/de/15-jahre-nki). The local climate policy database records all municipalities’ climate measures funded by the federal program since its rollout in 2008. In total, we have transformed this unstructured data into a curated dataset with 26,142 entries from 5,033 municipalities. The dataset stores variables on the type of action, project start and end date, and the amount spent on this project.

Next to this top-down approach, studying climate policy-making requires data on feedback effects between the public and the decision-making process. We account for these factors with two additional data sources:

First, German municipalities were strongly driven by bottom-up climate activism during the study period^12^. The discussion about the pros and cons of declaring a local climate emergency has likely been the most salient climate issue at the local level during the study period. Thus, our dataset integrates comprehensive hand-coded data on climate emergency motions submitted to the local council^13^. These motions were submitted by civil society groups and parties in many German municipalities in 2019 and subsequent years to accelerate local climate action. The finalized dataset records all incidents where motions to declare a climate emergency have been submitted to the local council, whether the municipality approved or declined the motion, and the date of the decision.

Second, research has shown how climate policy-making can have feedback effects on citizens’ policy preferences and voting behavior^14^. There is an increasing demand for georeferenced survey data which is linkable to policy output and outcome measures. To account for these research interests, we offer a flexible workflow to link the policy database to georeferenced public opinion data from large-scale, probabilistic survey programs in Germany. In particular, we have developed linking workflows to the georeferenced survey program GESIS Panel^15^ based on synthetic datasets derived from these products. These survey programs, hosted by the “GESIS - Leibniz Institute for the Social Sciences” in Germany, offer access to crucial items on environmental and climate attitudes and behavior, political and institutional trust, policy preferences, and voting behavior. These panel programs have a fine-grained spatial and temporal resolution. However, georeferenced survey data from respondents’ addresses is sensitive. Using location data from individual-level observations poses a significant risk of re-identifying survey respondents. Spatial data raises serious data protection concerns. To address these concerns, research projects that handle sensitive geocoordinates usually store the geometric information and the anonymized survey data in separate, access-controlled locations. Accordingly, external researchers cannot simply click a download button to retrieve this data. Instead, research data centers-such as those at GESIS-offer secure access facilities where researchers can work with the data on-site or via a secure remote client (see https://www.gesis.org/en/services/processing-and-analyzing-data/analysis-of-sensitive-data/secure-data-center-sdc/). These facilities typically restrict access to the internet and other external resources, and researchers often encounter the data for the very first time only after entering such an environment. It is there that they must build their entire data preparation and analysis pipeline, often under tight time constraints and with little opportunity for iteration.

Linking such data to databases, as in this paper, requires pre-processing and data preparation steps that cannot be conducted solely during on-site visits. Furthermore, the reproducibility of the analyses suffers. One solution to address these issues is to provide synthetic datasets that replicate the geographic structure of the original data. All derived attributes of linking should nearly mirror the distribution, like conducting the linking with the original data. To reproduce the linking workflow presented in this paper, we provide a synthetic dataset of the GESIS Panel geocoordinates comprising 1 sq km grid centroids. Our workflows for linking the policy database to these survey programs offer users easy, flexible data integration options that facilitate otherwise complex and burdensome data preparation before analyzing sensitive data. Our dataset contains synthetic georeferenced survey data that simulates real survey data without revealing sensitive information.

We synthesized the original georeferenced survey data in 4 steps: 1). We segmented the dataset by the 16 German federal states and identified how many municipality types according to the regional statistical spatial typology (RegioStaR) (see https://bmdv.bund.de/SharedDocs/DE/Artikel/G/regionalstatistische-raumtypologie.html) are in each state; 2). We counted how many respondents are located in each of these typologies; 3). We created a sampling frame of potential municipalities from which we can draw a sample; 4). We sampled as many 1 sq km grid centroids as in the original data based on their population density. Several additional measures were taken to ensure the anonymity of the original survey respondents, such as reusing only municipalities from the original data with at least 100,000 inhabitants, ensuring that at least 10 municipalities could be drawn from each spatial typology, and row-wise shuffling of all geocoordinates. We also deleted cases whose geographic distance between the synthetic and original data is less than 100 km. Details about this procedure can be found in the appendix as well as the public repository of the R package for synthesizing the data (see https://github.com/StefanJuenger/geosynth).

In addition to these micro-level indicators, we further combine the policy data with municipality-level context variables to support the study of local decision-making. To also accommodate the diverse usage behaviors and needs of researchers for these data types, we provide workflows in the statistical language R^16^ that enable flexible integration rather than static predefined variables. We argue that the impact of and on climate change must be considered. The theoretical expectation is that mitigation and adaptation efforts are systematically associated with climate indicators. Climate measures are expected to reduce greenhouse gas emissions in the long term and may also have additional benefits, such as reducing local pollution. At the same time, municipalities that experience extreme weather patterns might be more inclined to adopt climate policies. From an agency perspective, advocacy groups might strategically lobby municipalities that experienced such weather variations to submit climate emergency resolutions, and the general public might be more driven towards climate protection after experiencing local extreme weather.

To capture these mitigation and adaptation variables, we access data from national and European atmospheric and climate monitoring institutions. In particular, the German Weather Service (DWD), the Copernicus data services on Climate Change (C3S) (https://climate.copernicus.eu/) and Atmosphere Monitoring (CAMS) (https://atmosphere.copernicus.eu/) are integrated. Given that researchers require custom indicators tailored to their specific research question, we have created the R package gxc (https://github.com/denabel/gxc) to automate the linkage between these data sources and LOCC-GER. It offers users high flexibility in choosing indicators, as well as in spatial and temporal coverage and resolution. To exemplify and validate these indicators, we have created and provided a sample version of monthly weather indicators for temperature, precipitation, and wind based on data from the German Weather Service (DWD).

## Data Records

Given researchers’ need for up-to-date data and customizable indicators tailored to the specific operationalization of their research questions, we offer comprehensive workflows for flexible data integration with key context factors, such as climate indicators and public opinion. Furthermore, LOCC-GER is designed as a living dataset with annual updates to the policy database and aims to broaden the geographical coverage to other European countries. Version v1.0_2026 of the policy database^17^, including the data integration scripts and documentation, are stored in a OSF repository at https://doi.org/10.17605/OSF.IO/4EBXC. To ensure transparency and increase flexibility for users to adjust data processing to their own demands, we primarily store raw data points and provide extensive scripts for data processing and linking. A documentation file in the repository documents sources, coding schemes, and provides tutorials for transforming the raw data into the final output. Given that the policy data is in German, the documentation includes a table for translating all variables into English. The accompanying R scripts can directly link the database to the previously mentioned indicators. Since georeferenced survey data are highly sensitive, the public opinion data can only be accessed via GESIS’s secure data facilities. However, the availability of our synthetic survey data allows researchers to plan data processing, integration, and analysis in advance. LOCC-GER allows the full set up of a registered report and analysis scripts before accessing the original data.

The repository stores the main datasets (./data/) separated for the local climate policy (./data/local_policy), climate emergency data (./data/climate_emergency) and the synthetic GESIS Panel data (./data/gesis_panel). The accompanying code scripts (./scripts/) are structured in a similar way to represent preprocessing and linking of the datasets.

## Technical Validation

While our public opinion and policy data are derived from established and reliable sources, three data sections require further investigation: 1. the synthetic dataset of georeferenced survey data, 2. the climate activism data, and 3. the derived weather and climate variables from the German Weather Service (DWD).

Overall, our validation approach corroborates the strategy of synthesizing the GESIS Panel data. For this purpose, we linked the original and synthetic data to several geospatial datasets listed in Table 1, which represent typical and heterogeneous covariates in this research domain and are accessed from official German authorities. For example, for the case of precipitation data, all individual coordinates were linked to a count of extreme days of precipitation between March and May 2022, as defined as a positive deviation from the 90% quantile value of a 10-year baseline from 2011 to 2021. We also calculated the mean of a buffer zone of 5 km around the geocoordinates. Table 2 provides insights into the descriptive statistics of all derived attributes and the differences between these two datasets.

**Table 1:** Overview of indicators, data sources, and spatial linkage procedures for the validation of the synthetic georeferenced survey data.

Indicator	Data Source	Spatial Reference / Construction
Allocations for investment promotion measures	Indicators and Maps on Spatial and Urban Development in Germany and Europe (INKAR), Federal Institute for Research on Building, Urban Affairs and Spatial Development (BBSR)	Municipality-level indicator measuring public allocations for investment promotion measures. Values were linked to observations using municipal boundaries.
Days of extreme mean air temperature	German Weather Service (Deutscher Wetterdienst, DWD)	Number of days with extreme mean air temperature during the previous spring season (March–May) compared to the average of the preceding ten years. Values were calculated within a 10,000 m buffer around each observation.
Days of extreme precipitation	German Weather Service (Deutscher Wetterdienst, DWD)	Number of days with extreme precipitation during the previous spring season (March–May) compared to the average of the preceding ten years. Values were calculated within a 1,000 m buffer around each observation.
E-Charging Stations in 1000m	Federal Network Agency for Electricity, Gas, Telecommunications, Post and Railway (Bundesnetzagentur)	Count of publicly accessible electric vehicle charging stations located within a 1,000 m buffer around each observation.
Residential buildings with renewable heating	Indicators and Maps on Spatial and Urban Development in Germany and Europe (INKAR), Federal Institute for Research on Building, Urban Affairs and Spatial Development (BBSR)	District-level (Kreis) indicator representing the share of residential buildings equipped with renewable heating systems. Values were linked to observations using district boundaries.

**Table 2:** Descriptive statistics and percentage differences between original and synthetic datasets for selected spatial indicators.

Data Type	Minimum	Maximum	Mean	Median	SD	N Missing
**Allocations for investment promotion measures**						
Original Data	−3.58	948.00	127.00	97.90	119.00	1199
Synthetic Data	0.00	1029.00	124.00	97.10	125.00	1376
Difference in %	−100.00	8.49	−3.06	−0.85	5.32	14.80
**Days of extreme air temperatures**						
Original Data	7.95	29.70	17.70	16.70	5.35	0
Synthetic Data	7.95	29.80	18.30	18.20	5.40	0
Difference in %	-0.02	0.16	3.23	8.91	0.87	0
**Days of extreme precipitation**						
Original Data	4.19	23.50	13.50	13.50	3.28	0
Synthetic Data	4.60	23.50	13.60	13.50	3.05	0
Difference in %	9.91	−0.02	0.88	0.25	−7.02	0
**E-Charging Stations in 1000m**						
Original Data	0.00	542.00	9.30	3.00	22.90	0
Synthetic Data	0.00	568.00	9.72	3.00	25.20	0
Difference in %	0.00	4.80	4.55	0.00	9.99	0
**Residential buildings with renewable heating**						
Original Data	20.20	100.00	65.90	69.20	16.10	3
Synthetic Data	20.20	94.80	64.20	67.80	17.20	0
Difference in %	0.00	−5.24	−2.49	−1.98	6.62	−100

It is expected to find differences in these statistics between the original and synthetic data. However, they must provide a trade-off between the anonymity of the original data and the resemblance of the synthetic data. To assess the impact of synthesis, we left the geospatial data attributes at their original measurement levels, but varied the aggregation level of the linkages as described in Table 1. We also introduced one indicator with a large amount of missing data to investigate how linkages with inflated amounts of missings bias the results. Indeed, we find differences between the original and the synthetic data, and by looking at absolute numbers, they may not be negligible. For example, the difference in the amount of missing data for the indicator “Allocations for investment promotion measures” is 14.8% points. While this indicator includes negative values in the original data, it does not in the synthetic data. Nevertheless, in many cases, the differences affect the extremes of the distributions and may arise from small numbers of observations. It is likewise important to investigate the overall distribution of the variables after they were linked.

**Fig. 4:**
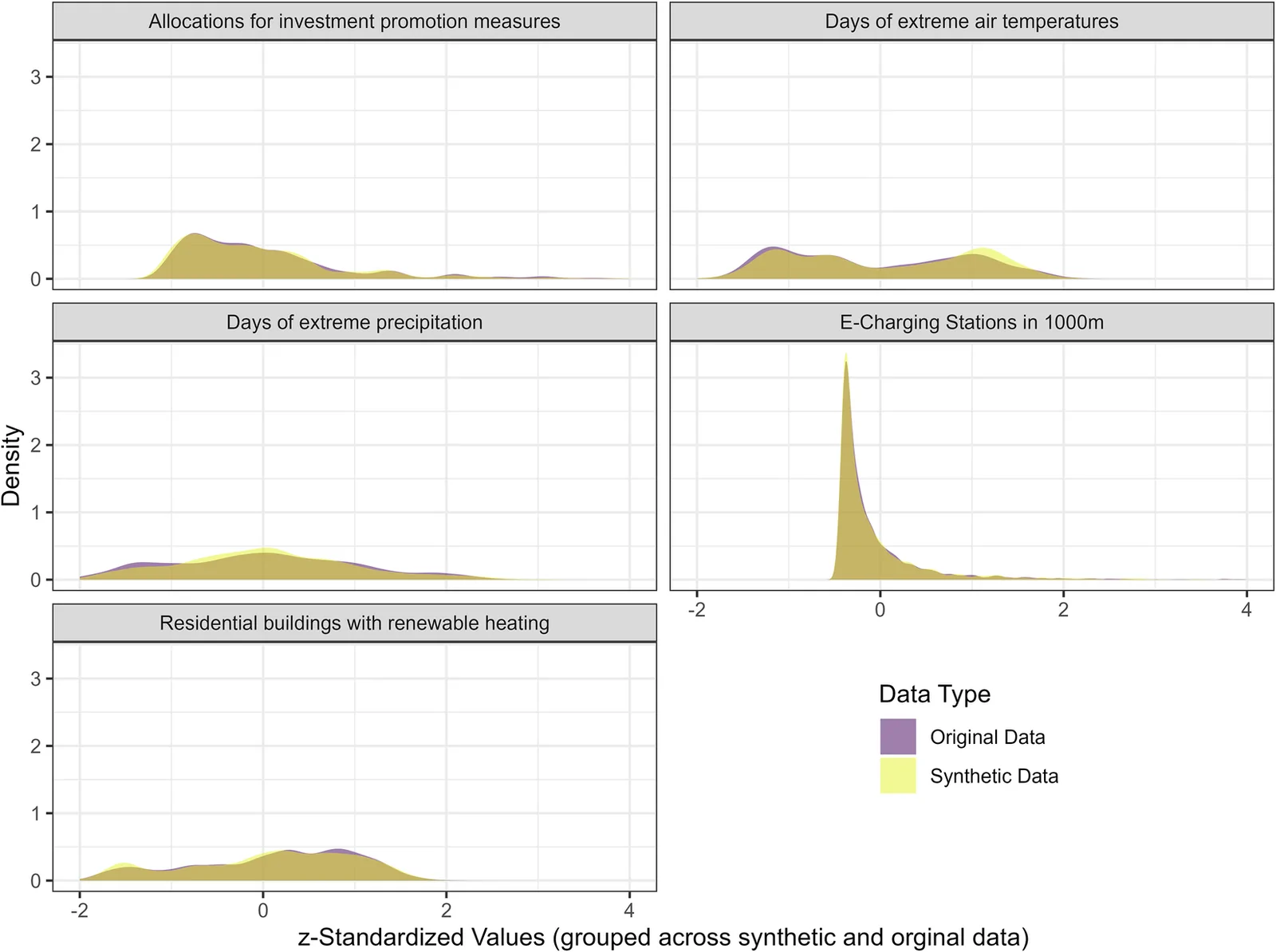
Densities of the linked geospatial data indicators across the original and synthetic data.

As shown in Fig. 4, there are slight differences in the variable density within each dataset, yet both distributions follow a comparable pattern. For this exercise, we z-standardized the geospatial indicators across the synthetic and original datasets. Strikingly, the indicators display differential distributions after the linkage that are equally captured by the original and synthetic datasets. Researchers aiming to link the original data in an on-site setting, as described above, could first rely on synthetic data to assess the feasibility of the linkages. After all, resembling the overall distributions of linked indicators may be more important than the absolute number of extreme values in the very same distribution.

Regarding the data on climate activism, the dataset integrates comprehensive, hand-coded data on climate emergency motions submitted to the local council. Extensive desk research was conducted from June to August 2024 to identify all submitted motions. Several sources provided by activist groups and observers (a. https://www.klimabuendnis-hamm.de/klimanotstand-in-jedem-rathaus/, b. https://kommunalwiki.boell.de/index.php/Klimanotstand, c. https://de.wikipedia.org/wiki/Liste_deutscher_Orte_und_Gemeinden,_die_den_Klimanotstand_ausgerufen_haben) were cross-checked with municipal press releases to ensure the validity of the data. To validate the quality of the climate emergency data, a second coder replicated the coding procedure between April and May 2026. The documentation file in the repository documents the procedure and selection criteria for diverging entries.

As described above, we have used DWD data to link our data to climate variables. The DWD provides hydrometeorological grid data for the whole of Germany as part of their Hyras database^18^. These data are derived from single-station weather data, projected to a grid level, and adjusted for different height profiles and urban heat islands. Still, orographic effects are evident in the data, which include temperatures on a 5 sq km grid and precipitation used to validate the synthetic survey geocoordinates on a 1 sq km grid. For this reason, we decided to conduct another validation of the data using the raw station data and the derived grid-level data.

**Fig. 5:**
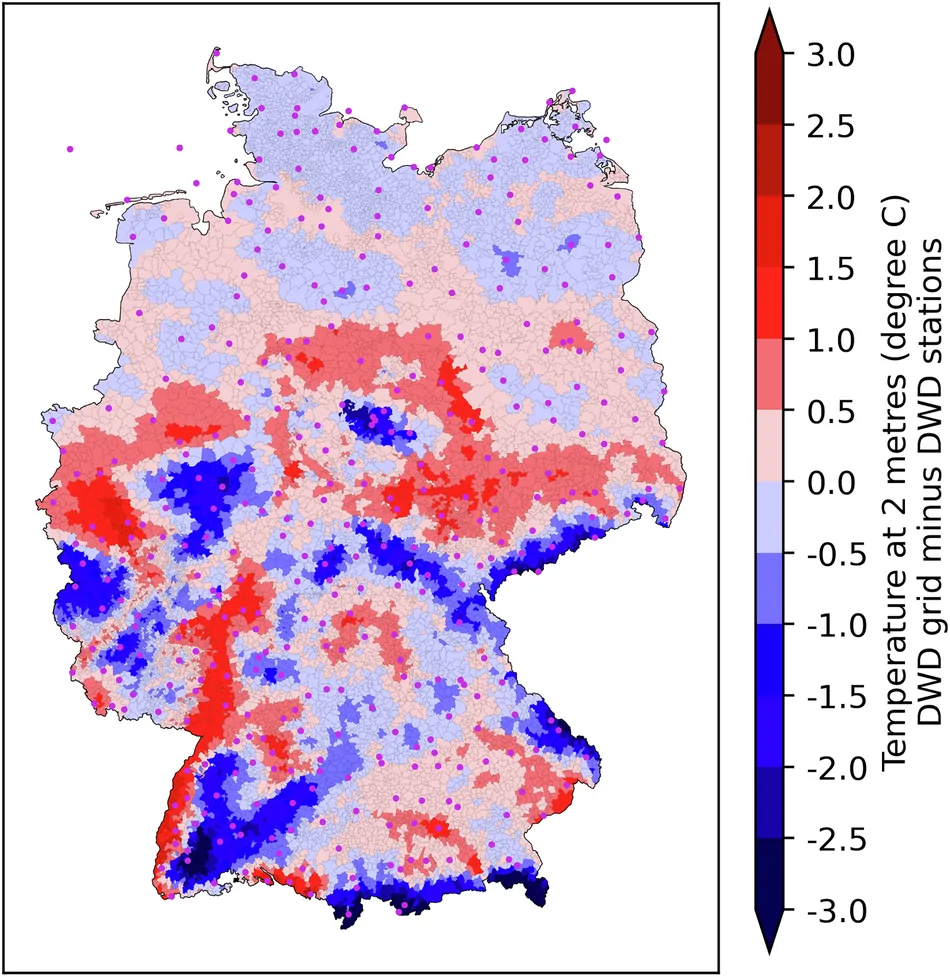
Map of the Differences of Temperatures on the Municipality Level Based on Grid-Level and Station-Level Data. Dots show the locations of individual weather stations.

**Fig. 6:**
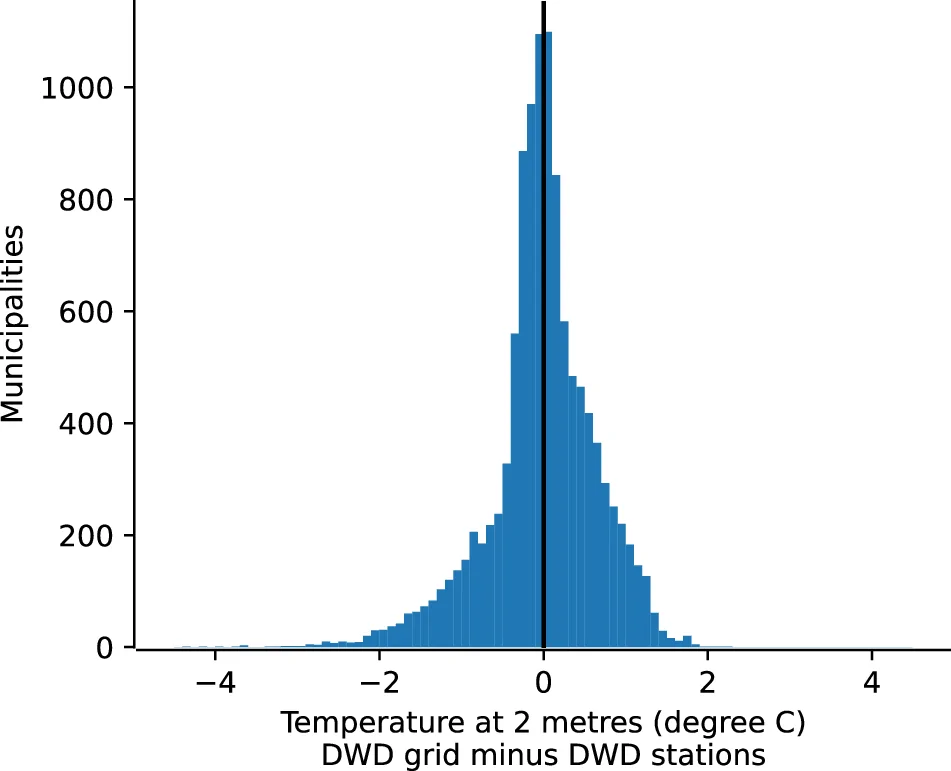
Histogram of the Difference in Temperatures on the Municipality Level between the Grid-Level and the Station-Level Data.

To better understand the impact of potential differences between these data, we aggregated both data sources to the municipality level. For the grid-level data, we derived simple mean values from all grids that intersected the municipality polygons; for the station-level data, we calculated a mean of the individual station values using inverse-distance weighting with a distance cutoff of 100 km. Fig. 5 shows a map of all German municipalities and the differences in mean temperature between the aggregated gridded data and the aggregated raw station data. There are significant differences in the absolute values. However, when plotting these differences as a histogram in Fig. 6, we observe approximately symmetrical deviations in either direction. We conclude that the orographically adjusted grid-level data that we use in this study is preferred, but that station-level data may also provide robust - albeit less accurate - estimates of climate variables in settings where adjusted grid-level data are missing.

## Usage Notes

As one of the most extensive and detailed datasets on local climate policy, LOCC-GER supports social and economic research on broad issues of the evaluation of evidence-based policy-making, place-based politics, and policy innovation, sequencing, or diffusion. For example, researchers could explore 1. policy adoption, diffusion and feedback patterns across particular sectoral policies or regional areas, 2. how local policy-making adjusts to pressures from civil society or environmental factors, 3. how local public opinion reacts to the implementation or neglect of local climate policy-making, 4. how local environmental factors such as pollution change due to local climate policy-making, or 5. accordingly, to evaluate the effectiveness of local climate policy-making. Importantly, our synthetic public opinion data enables researchers to set up a custom-made linking strategy between the policy database and one of the most relevant and long-running German panel studies. We also see high potential in linking this dataset to other georeferenced datasets on local climate city networks^19^, georeferenced social media data on climate opinions^20^, or electoral outcomes in local and regional elections^21^.

## Data Availability

All datasets are stored in the OSF repository https://doi.org/10.17605/OSF.IO/4EBXC.

## Code availability

All code to generate LOCC-GER and to reproduce the visualizations in this report, are publicly available at the OSF repository https://doi.org/10.17605/OSF.IO/4EBXC. The current version of the geosynth package for creating synthetic georeferenced survey datasets can be accessed at https://github.com/StefanJuenger/geosynth. Our gxc package for flexible linking of LOCC-GER with weather and climate data from national and European monitoring services is available at https://github.com/denabel/gxc.
